# Trivialities in metabolomics: Artifacts in extraction and analysis

**DOI:** 10.3389/fmolb.2022.972190

**Published:** 2022-09-08

**Authors:** R. Verpoorte, H. K. Kim, Y. H. Choi

**Affiliations:** ^1^ Natural Products Laboratory, Institute of Biology Leiden, Leiden University, Leiden, The, Netherlands; ^2^ College of Pharmacy, Kyung Hee Univeristy, Seoul, South Korea

**Keywords:** artifacts, metabolomics, extraction, solvents, decomposition

## Abstract

The aim of this review is to show the risks of artifact formation in metabolomics analyses. Metabolomics has developed in a major tool in system biology approaches to unravel the metabolic networks that are the basis of life. Presently TLC, LC-MS, GC-MS, MS-MS and nuclear magnetic resonance are applied to analyze the metabolome of all kind of biomaterials. These analytical methods require robust preanalytical protocols to extract the small molecules from the biomatrix. The quality of the metabolomics analyses depends on protocols for collecting and processing of the biomaterial, including the methods for drying, grinding and extraction. Also the final preparation of the samples for instrumental analysis is crucial for highly reproducible analyses. The risks of artifact formation in these steps are reviewed from the point of view of the commonly used solvents. Examples of various artifacts formed through chemical reactions between solvents or contaminations with functional groups in the analytes are discussed. These reactions involve, for example, the formation of esters, *trans*-esterifications, hemiacetal and acetal formation, N-oxidations, and the formation of carbinolamines. It concerns chemical reactions with hydroxyl-, aldehyde-, keto-, carboxyl-, ester-, and amine functional groups. In the analytical steps, artifacts in LC may come from the stationary phase or reactions of the eluent with analytes. Differences between the solvent of the injected sample and the LC-mobile phase may cause distortions of the retention of analytes. In all analytical methods, poorly soluble compounds will be in all samples at saturation level, thus hiding a potential marker function. Finally a full identification of compounds remains a major hurdle in metabolomics, it requires a full set of spectral data, including methods for confirming the absolute stereochemistry. The putative identifications found in supplemental data of many studies, unfortunately, often become “truly” identified compounds in papers citing these results. Proper validation of the protocols for preanalytical and analytical procedures is essential for reproducible analyses in metabolomics.

## 1 Introduction

In the past 2 decades metabolomics has rapidly developed as an important tool in studying various biological and medical questions. The aim of metabolomics is the qualitative and quantitative analysis of all small molecules present in biological samples. By comparing the metabolome of organisms under different conditions, information can be obtained about the regulation of the metabolic network and the potential biological role of specific compounds. The analysis of the metabolome is complicated because a wide spectrum of compounds with totally different physico-chemical properties are present in a wide range of concentrations. That makes the measurement of all small molecules in an organism in one operation the major challenge for the 21st century’s (aL)chemistry.

The analysis of the small molecules is done by means of chromatographic separations (TLC, LC or GC) coupled to UV-, mass- (MS), or nuclear magnetic resonance (NMR) spectrometry. Alternatively the analysis is done directly by MS/MS or NMR. Each of these methods has its own advantages and disadvantages. These aspects will be dealt with in other chapters. In this chapter we want to focus on some basic problems that one should keep in mind when developing a metabolomics analysis.

The goal of this chapter is to highlight the problems of artifact formation in the preanalytical and analytical procedures. Artifacts are described as the compounds that are not present in an intact metabolome but are formed in the process of harvesting, drying, grinding, extracting, and preparing samples for analysis, and during the separation and detection phase of the analysis.

Artifacts can be formed by a reaction of an analyte with the solvents itself or with contaminants in solvents. In the books on Chromatography of Alkaloids (Baerheim Svendsen and Verpoorte 1983; Verpoorte and Baerheim Svendsen. 1984) we have reviewed some of these problems in connection with alkaloids. In other papers we discussed some basic aspects of metabolomic analyses (Verpoorte et al., 2008) and artifact formations with solvents (Maltese et al., 2009). The present chapter summarizes these earlier papers as well as some more recent examples. This information should be useful to have at hand in a book on metabolomics. This is not rocket science, but it concerns trivial things that people forget when running automated instrumental analyses. For newcomers not trained in natural products chemistry or analytical chemistry this may be new information. At least when reviewing papers in this field we are often surprised by the ignorance about basic methods to prepare the sample (extract) for the final high-tech analysis.

## 2 Solvents for extraction and chromatography

To discuss artifact formation of all known compounds would be a huge task, not to speak about all unknown compounds. Instead, we will focus on the most common solvents and their known contaminations (Table 1) that may play a role in generating artifacts. For a comprehensive survey of contaminations in solvents and reagents is referred to Middleditch (1989) and Venditti (2020). In the former publication, the molecular weights and mass spectra of common solvents, additives, and contaminations like plasticizers, paper whiteners, rubber constituents, and antioxidants, are described. In addition, there are review papers on specific group of metabolites. Capon (2020) reviewed the artifact formation for marine natural products. The possible mechanisms of the artifact formation are discussed in depth. They vary from simple esterification, solvolysis and oxidation to highly complex chemical rearrangements. Artifact formation of various terpenoids was reviewed by Hanson (2017). Dehydration, rearrangements, and oxidation, among others, cause formation of artifacts from all kinds of terpenes. Xu and colleagues (2020) reported methods to predict potential artifacts by reactions with methanol or oxidation. Venditti (2020) particularly discussed monoterpenoid artifacts.

**TABLE 1:** The example of impurities and reactions of the solvents most commonly used in phytochemistry.

Solvent class	Solvents	Contaminations	artifacts
Alcohol	Methanol, ethanol, propanediol, glycol, glycerol	Aldehydes	Esters, acetals, hemiacetals, carbinolamines
Ethers	Diethyl ether, tetrahydrofuran	Peroxides, aldehydes, alcohols	N-oxides, carbinolamines, esters, acetals, hemiacetals
Esters	Ethyl acetate	Acetaldehyde	*Trans*-esterifications, Esters, acetals, hemiacetals, carbinolamines
Acetonitrile	Acetonitrile		Acetamide, BHT, dichlorobenzene, glutatonitrile, succinonitrile
Chloroform	Chloroform	Phosgene, CH_2_BrCl, CH_2_Cl_2_	Quaternary amines
Dichloromethane	Dichloromethane	CH_2_BrCl, CNCl	Quaternary amines cyanides
Aromatic hydrocarbons	Toluene		Various Hydrocarbons

## 3 Stability of analytes

Papers describing stability of compounds under different conditions often claim that a compound is not stable, but there are very few papers that really have known standards as controls. In our experience terpenoid indole alkaloids are not very stable. People working with isoquinoline alkaloids claimed that these alkaloids were very labile. However, working on both types of alkaloids, it was obvious that most isoquinoline alkaloids were more stable than the indole alkaloids. Apparently, there are more feelings, than there is understanding. It is difficult to predict solubility and stability of pure compounds. In general, the experience is that light and heat are an important factors. Keep the compounds, when dissolved, always in the dark, at the lowest possible temperature. This also means that extractions using Soxhlet equipment are extremely detrimental for the analysis of the true metabolome of an organism. Alcohols are in general the best solvents to store compounds and extracts. In general, the stability of compounds in the halogenated solvents is low, compounds like the anhydronium indole alkaloids serpentine and alstonine do not survive dissolving in chloroform (Verpoorte and Sandberg 1971; Baerheim Svendsen and Verpoorte 1983). Also reserpine and related indole alkaloids are rapidly oxidized in chloroform (Wright and Tang, 1972). The pH does play a role, though for each compound the optimum can be different. Moreover in mixtures there can be differences, e.g. by the presence of natural antioxidants in extracts. In NMR-based metabolomics the use of fully deuterated solvents, like methanol and D_2_O, may cause the replacement of certain protons with deuterium, e.g. in aldehydes and ketones via a keto-enol equilibrium. For example, naringenin has been shown to have two phloroglucinol protons to be completely exchanged in the presence of a solvent with a deuterated hydroxyl group (Verpoorte et al., 2008). Finally, one should also keep in mind that the stationary phases used in chromatography also play a role. For example, Pauli and co-workers (Tang et al., 2021) showed that silica affects the oxidation of prenyl groups in various natural products.

## 4 Reactions of solvent or solvent’s impurities with analytes

Many preanalytical protocols have been reported for extraction and sample preparation. Here we will confine us to the solvent itself as a chemical that may react with analytes and illustrate this with some examples.

**FIGURE 1:**
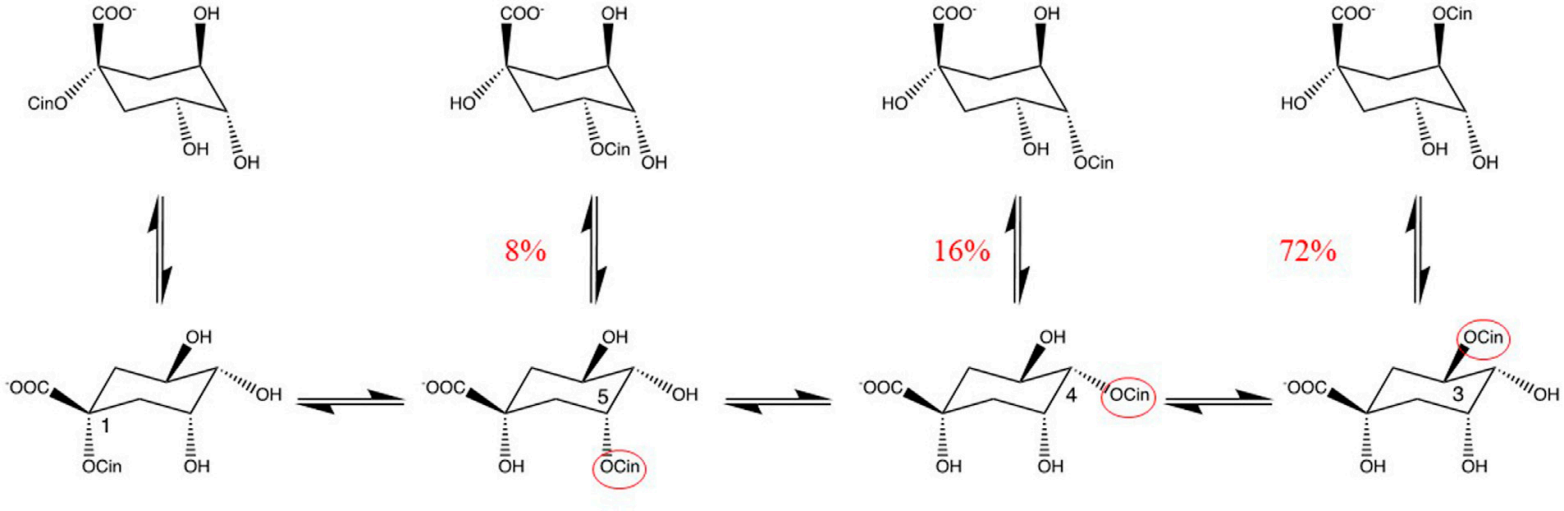
Isomerization of chlorogenic acids. Intramolecular migration of the cinnamoyl-group (cin) in pure chlorogenic acid (100% pure 3-cinnamoylquinic acid) when 3 min in phosphate buffer pH 7, at 90°C (Hanson, 1965). Percentages given are quantities relative the pure chlorogenic acid. cin = cinnamoyl.

***Alcohols*** Alcohols are often used in extraction. Methanol is commonly used to extract biological samples for metabolomic analysis. However, methanol is toxic and thus for applications in food, medicines or cosmetics, ethanol, 1,2-dihydroxypropane, glycol and glycerol are preferred. In liquid chromatography methanol is often used as component of the mobile phase. The reactive site of alcohols is a hydroxyl group. The reaction of carboxyl group(s) of analytes with alcohols may yield esters. Even, inter- and intra-molecular *trans*-esterifications may occur. The fast isomerization of chlorogenic acid is a good example of an intramolecular *trans*-esterification of the cinnamoyl group. Within 3 min at 90°C in water pH 7, about 28% of the pure chlorogenic acid was found to be isomerized (Hanson, 1965; Clifford et al., 1989) (Figure 1). This example shows that one should be very careful in drawing conclusions from any changes in the levels of these compounds, which are ubiquitous in plants.

Methanol and ethanol have been reported to react with fatty acids to generate esters during extractions (Lough et al., 1962; Johnson et al., 1976; Xu et al., 2020). Brondz and colleagues (2007) reported esterification of the carboxylic acid group in β-carboline alkaloids. The effect of methanol was studied in more detail by Xu and coworkers (2020). They developed a chemometric tool to predict potential artifacts by reactions with methanol or oxidation. Some benzylisoquinoline alkaloids and caffeic acid derivatives were used as examples. Sauerschnig and colleagues (2018) reported many other examples of artifact formation with methanol. By using deuterated methanol, they showed by LC-MS that 8% of the more than 1,100 detected metabolites were artifacts containing a deuterated OMe group.

**FIGURE 2:**
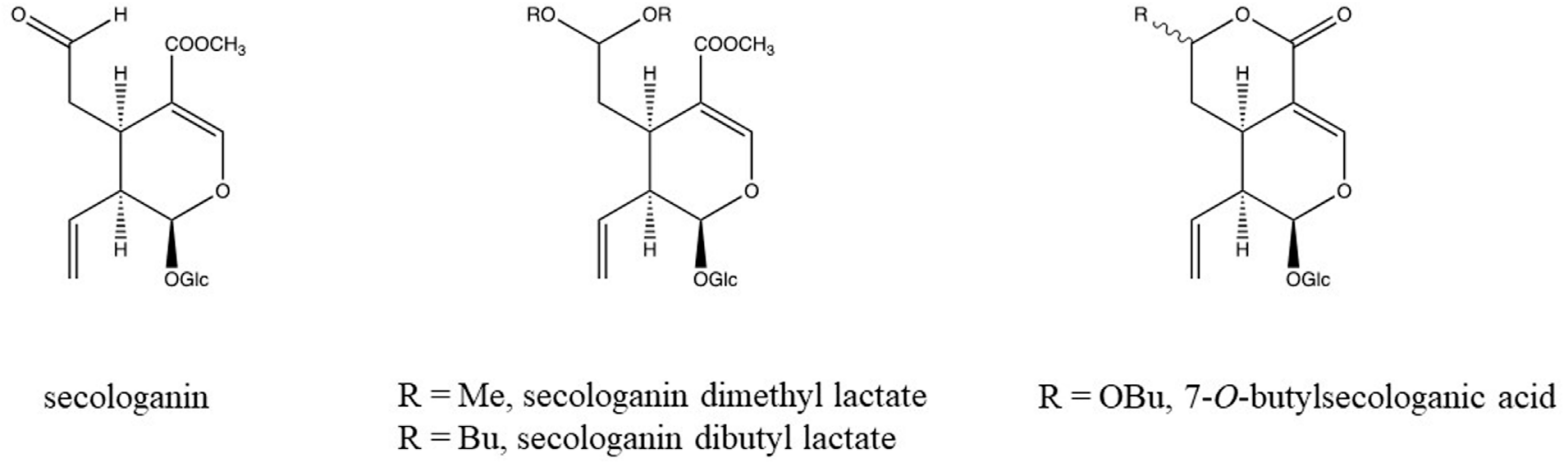
Chemical structure of secologanin and artifacts formed during isolation in alcoholic solvents (Verpoorte unpublished results, Tomassini et al., 1995).

**FIGURE 3:**
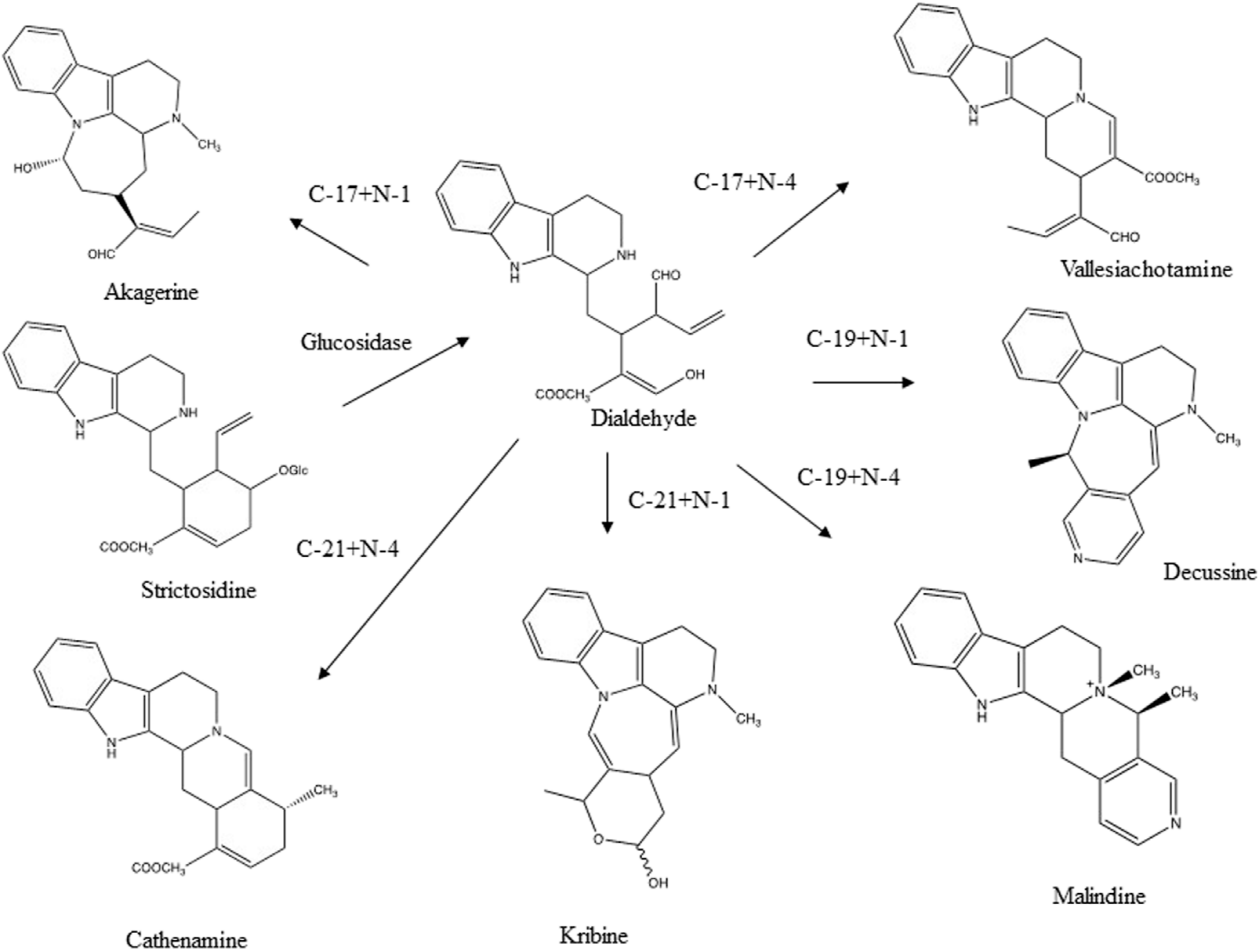
Various skeletons of terpenoid indole alkaloids formed through the intramolecular reaction of the aldehyde group and an amine function after glucolysis of strictosidine (Verpoorte, 2000).

Another reaction concerns aldehyde- and keto-groups. They may react with alcohols to yield hemiacetals and acetals. In chromatography a single pure compound may show several peaks due to these reactions. Secologanin is an example of such a compound (Figure 2). Both intra- and intermolecular reactions may be involved. Acetone may even form adducts with secologanin (Verpoorte unpublished results, Tomassini et al., 1995). Aldehyde- and keto-groups maybe involved in all kinds of internal rearrangements, like in the biosynthesis of terpenoid indole alkaloids, in which strictosidine is the precursor for a large number of pathways leading to different skeletons (Verpoorte, 2000) (Figure 3). The first reaction is the loss of a glucose. This leads to the opening of the acetal containing ring, in which the molecule unfolds to give two reactive aldehyde functions and two reactive amino groups. This opens the biosynthetic pathways leading to different structures. The reactions of an aldehyde- or keto-group with an amine or an hydroxyl group are important reactions to keep in mind as they are a major source for artifacts.

The alkaloid gentianine is an example of a non-natural alkaloid that is formed during extraction with an ammonia containing extraction solvent. The ammonia may react with the aldehyde group in the iridoid sweroside, yielding gentianine (Phillipson et al., 1974; Popov et al., 1988). Bunel and coworkers (2014) showed that 2-hydroxy-4-methoxybenzaldehyde reacts with ammonia to give an alkaloid like compound. Wenkert and co-workers (1965) reported the artifact formation of an abietane-type of diterpene when ammonia was used in the extraction.

**FIGURE 4:**
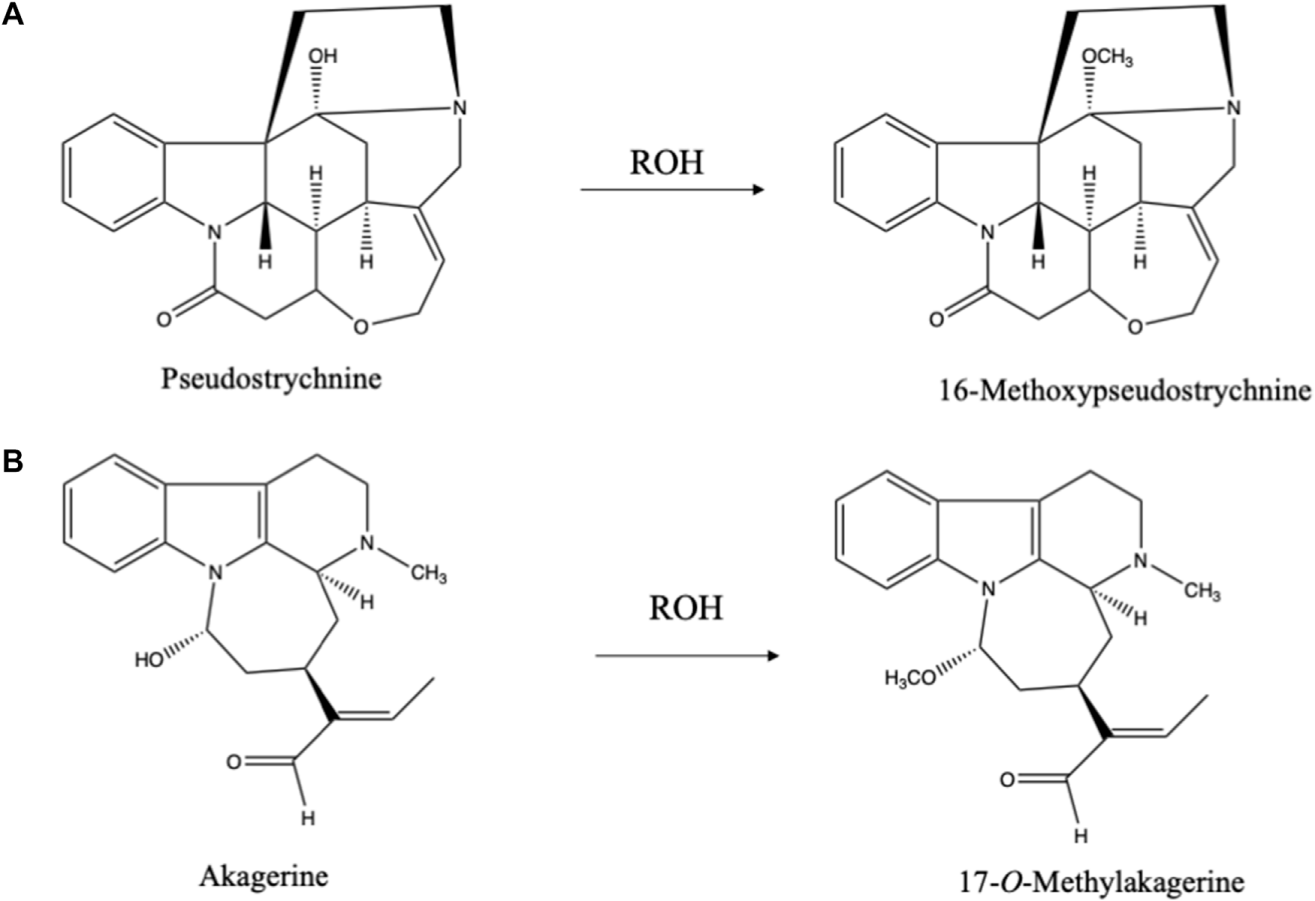
**(A)** Artifacts formed during isolation of pseudostrychnine in alcoholic solvent (Bisset et al., 1965). **(B)** Artifact formed during isolation of akagerine in alcoholic solvent (Rolfsen et al., 1978).

The hydroxyl group in carbinolamines easily reacts with alcohols (just like hemiacetals) yielding an *O*-Methyl derivative in case of methanol as extraction solvent. 16-Methoxypseudostrychnine (Bisset et al., 1965) (Figure 4A) and 17-*O*-methylakagerine (Rolfsen et al., 1978) (Figure 4B) are examples of such artifacts. In this connection the use of ethanol as extraction solvent has advantages. First of all, it is a greener solvent than methanol and is less toxic. Moreover, in case of any reaction with an alcohol during the extraction one will find an ethoxy group instead of a methoxy group. As ethoxy groups are rare in nature, this is an excellent method for for identifying potential artifacts.

A keto-group containing solvent like acetone, may form adducts with ammonia or amines that give alkaloid-positive color reactions, particularly when running preparative column chromatography on silica (Householder and Camp 1965). Alcohols may contain aldehydes and carboxylic acids as contaminations. One should keep in mind that commercial chloroform always contains 1–2% of ethanol. That means that the above-mentioned reactions also occur in chloroform solution (see below). In general, the experience is that dissolved in alcohols compounds are reasonably stable.

***Ethers*** Though ethers are rather inert in terms of reactivity if compared to alcohols, their major problem is the formation of peroxides. In the use of ethers, great caution is required when evaporating ethers because of high risks of explosions. These peroxides mediate artifact formation, as complex natural products can be oxidized by these peroxides. The most common one is the well-known *N*-oxidation of amines. The *N*-oxides formed may further react and cause ring openings, exemplified by the case of strychnine where the *N*-oxide rearranges into the hydroxy derivative (pseudostrychnine) (Figure 4A) (Bisset et al., 1965). Via this carbinolamine a ring can be opened. N-oxidation is a common step in the catabolism of alkaloids and nitrogen containing medicines. When choosing for diethyl ether as solvent, one should always check for the presence of peroxides.

***Esters*** The intramolecular *trans*-esterifications in chlorogenic acid and their analogues were mentioned above. Using esters (e.g. ethyl acetate) as solvent incurs the risk of *trans*-esterifications. The combination of ammonia and ethyl acetate may lead to crystallization of acetamide.

***Halogenated solvents*** For the extractions of medium polar compounds and for liquid-liquid purifications, halogenated solvents such as chloroform and dichloromethane are often used. For toxicity reasons dichloromethane is recommended to be used instead of the more toxic chloroform. Because of physico-chemical properties chloroform has some advantages in better dissolving medium polar compounds, and in particular alkaloids. In terms of artifacts both have serious disadvantages (Baerheim Svendsen and Verpoorte 1983). Besselièvre and coworkers (1972) asked the question “is dichloromethane a solvent or a reagent”. They found that the indole alkaloid tubotaiwine is rapidly converted to the quaternary dichloromethotubotaiwine when dissolved in dichloromethane. Strychnine, brucine (Phillipson and Bisset 1972; Verpoorte et al., 2008) and atropine (Vincze and Geven, 1978) have been reported to also give such quaternization of an amine function with dichloromethane. These quaternary dichlorometho alkaloids have lost the lipophilic properties of the tertiary alkaloids. Also, with chloroform these artifacts were formed, though at lower levels as they are formed with dichloromethane and dichlorobromomethane present in chloroform as contaminations (Phillipson and Bisset 1972). Hansen (1977) reported on the artifact formation through *N*-alkylation of amines. In case of strychnine and brucine, in addition to dichlorometho artifacts and N-alkylation, also *N*-oxides and the pseudo-strychnine and -brucine were formed in the chlorinated solvents

**FIGURE 5:**
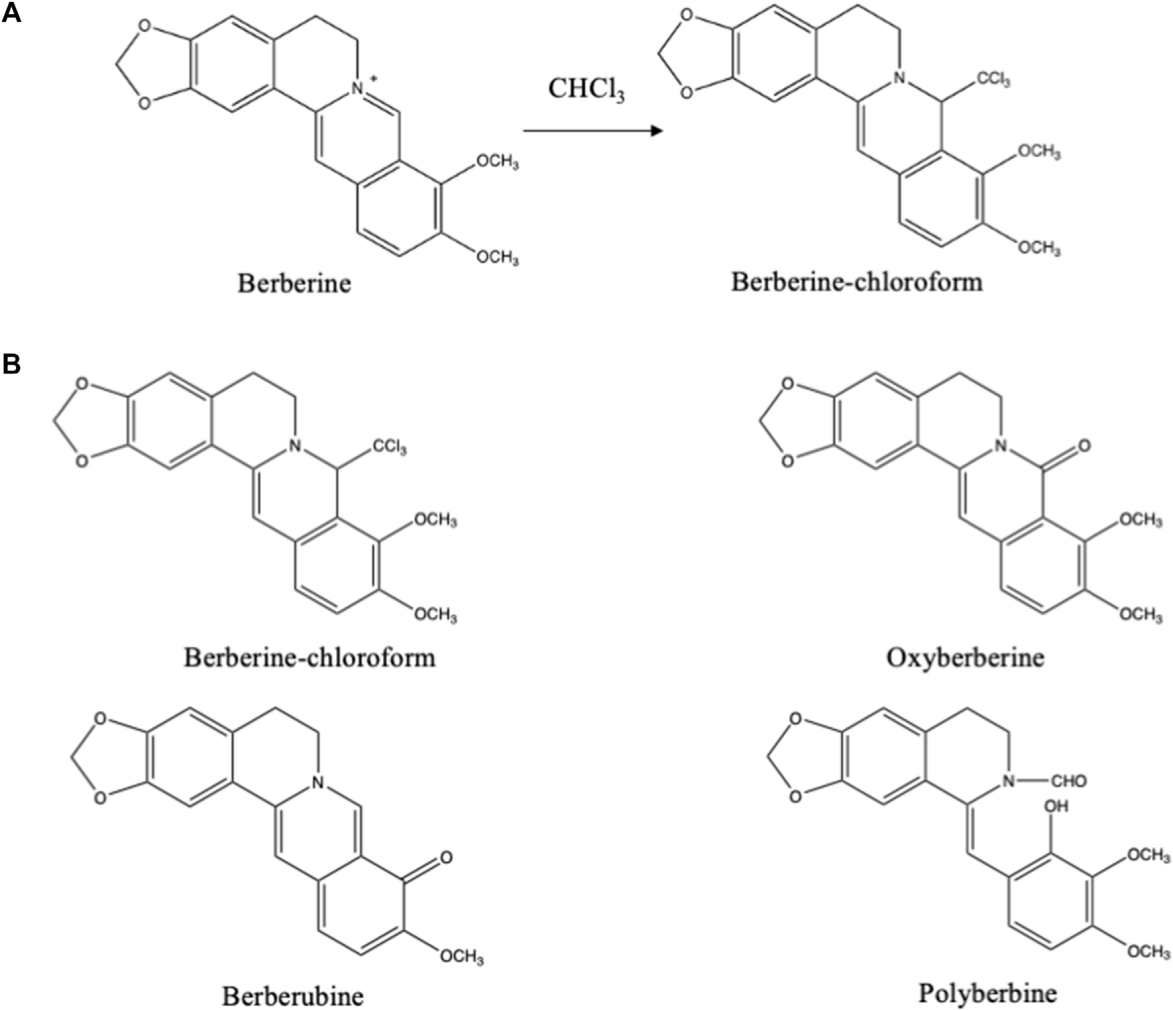
**(A)** Artifact formed from berberine during its isolation using chloroform (Miana, 1973). **(B)** Artifacts derived from berberine formed during column chromatography using chloroform:methanol (99:1) as eluting system (Shamma and Rahimizadeh, 1986).

In dichloromethane cyanogen chloride (CNCl) might be present in variable quantities as contamination (Franklin, et al., 1978). Primary and secondary amines may form nitriles with this impurity. In chloroform, however, no CNCl could be detected. Chloroform itself reacts with protoberberine type of alkaloids. Especially, during column chromatography using chloroform-methanol as mobile phase, a trichloro compound was formed from berberine (Figure 5A) and related alkaloids (Miana, 1973). Through oxidation other artifacts were formed from the these alkaloids (Figure 5B) (Shamma and Rahimizadeh, 1986).

**FIGURE 6:**
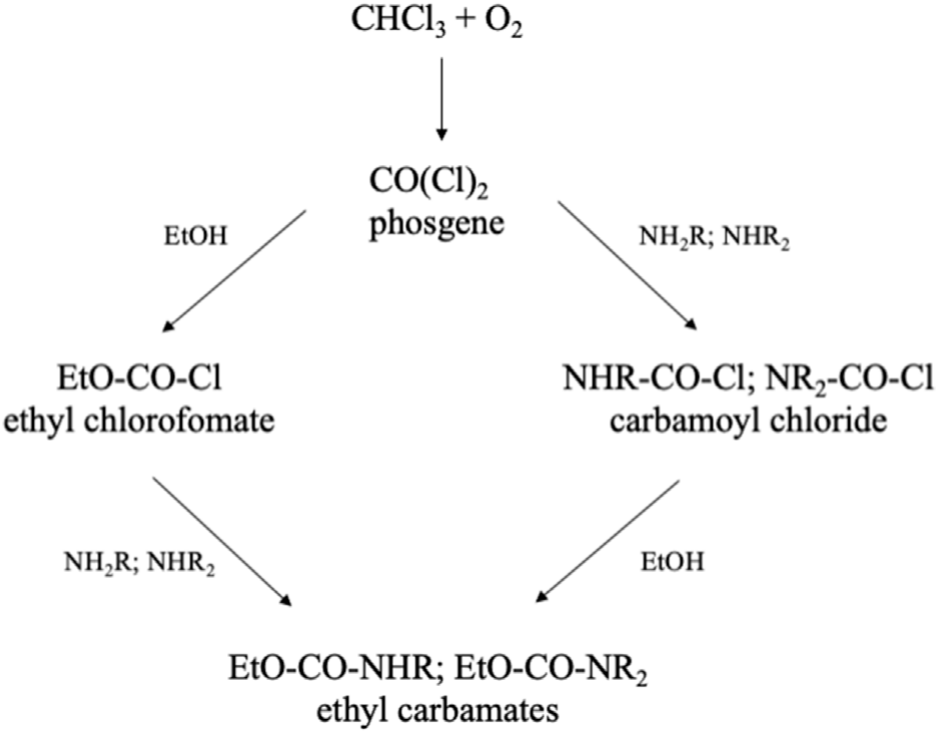
Formation of ethyl chloroformate in chloroform and possible resulting adducts (Siek et al., 1977; Cone et al., 1982; Maudens et al., 2007).

Another problem with chloroform is its oxidation in the light, yielding phosgene, a well-known chemical warfare gas. This highly reactive gas reacts with all kinds of compounds. To neutralize phosgene, chloroform always contains 0.5–2% of ethanol. Ethanol reacts with phosgene (Figure 6), and thus keeps the level of phosgene low, but still there will be some artifacts formed with analytes. In the analysis of normeperidine the ethyl chloroformate derivative of the target compound was detected (Siek et al., 1977). Cone and colleagues (1982) reported artifact formation in the analysis of metabolites of codeine, when chloroform was used to extract the alkaloids from biological fluids. A similar study was published for the extraction of anthracyclines (Maudens et al., 2007). The presence of ethanol in chloroform may also be the cause of artifacts as described above for alcohols. By distillation chloroform can be purified, but always some alcohol should be added after distillation.

## 5 Chromatography related problems

### 5a Identification of compounds

Pimms et al. (1995) made an estimation of the number of organisms on earth. The estimation was between 10 and 100 million organisms, among which 250.000 plant species. A search made at the end of last century in the NAPRALERT database (NAtural PRoducts ALERT, focused on natural products and their bioactivities) showed that of total plant biodiversity, around 15% of the species had been studied to some extent for secondary metabolites and only about 5% for one or a few biological activities (Verpoorte 1998, 2000; Verpoorte et al., 2006). In the Dictionary of Natural Products (2022) the present number of compounds is 328,000. If we assume that every species can make one unique compound, there must be millions of yet unknown compounds present in nature. Based on the number of genes in a plant we estimate that a plant may contain 20,000 to 50,000 different compounds with a very broad range of polarities and with a huge dynamic range.

With the high resolution of the state-of-the-art hyphenated metabolomics methods, we expect many new compounds to be reported in the coming years. Putative identifications of compounds are made through searching various databases with information about retention behavior, molecular weight, and MS-fragmentation patterns. Tools like the recently developed molecular networking are helpful in identification of known compounds, as well as in predicting the chemical structures of novel compounds (Aron et al., 2020). However, for proper identification of a compound and for structure elucidation of novel compounds, the mentioned information is not sufficient for a full identification. A complete set of spectroscopic data is needed to confirm the chemical structures and the stereochemistry. Unfortunately, in literature one may find publications with supplementary data with a long list of putative “identified” compounds, based on retention time, molecular weight, MS-fragmentation and comparison with existing databases. These “identifications” are later often cited in other papers as identified compounds, without further new evidence for the identification. One may consider such identifications also as artifacts generated by the automated analytical methods. To avoid doubt about identifications, recommendations have been made for the level of confidence of an identification from metabolomics data (Blaženovic et al., 2018). Whether it is acceptable to say that a component is with 90% confidence compound X, or similar “statistical” support, is in our view doubtful. At least for any marker molecule identified through metabolomics analysis, one should have hard spectral evidence for the identity.

### 5b Injection samples

Extracts must be dissolved at a certain point in the preanalytical processing for further separation or analysis. There are numerous solvents to extract or to redissolve extracts or molecules obtained from a biological sample. In the different analytical procedures, different procedures are needed. In the Liquid chromatography (TLC and HPLC) the extract must be completely dissolved in a proper solvent. For TLC the choice is based on the solubility of the extract, and on the ease of applying the sample on the plate, where after application the solvent has to be evaporated, before the development of the TLC plate. In case of HPLC the solvent of the sample for injection in the LC-system must be compatible with the column and the eluent. For example, in developing a protocol for a metabolomic analysis, the final solvent used to inject a sample in LC should be as similar as possible to the mobile phase in terms of polarity and pH, including the presence of compounds that may affect retention behavior (e.g. ion-pairing). Ion-pairing LC is used in the analysis of alkaloids, e.g. using long chain sulfonic acids as additive in the mobile phase. But also, ions like acetate, formate, trifluoroacetate, chloride, bromide, and iodide may act as ion-pairing agents, and in liquid-liquid extractions they may cause loss of analytes (Hermans and Verpoorte 1986). In case of large differences between injection solvent and mobile phase the retention of some analytes may be affected. Even the formation of multiple peaks for a single molecule may occur in case of a large difference. In the analysis of complex chromatograms this may easily be overlooked, resulting in errors in quantitation and identification. In case of GC the solvent for dissolving an extract should enable the derivatization required to volatilize the various components present in the metabolome. In case of NMR the NMR-solvent can be used to extract the biomaterial, the reduction in workload is a major advantage of NMR. In case of mass spectrometry solid extracts or biomaterials can be used when the equipment offers this option. The analytical method applied determines the necessary preanalytical procedures. Obviously there will be differences between the various analytical procedures for risks of artifacts formation.

**FIGURE 7:**
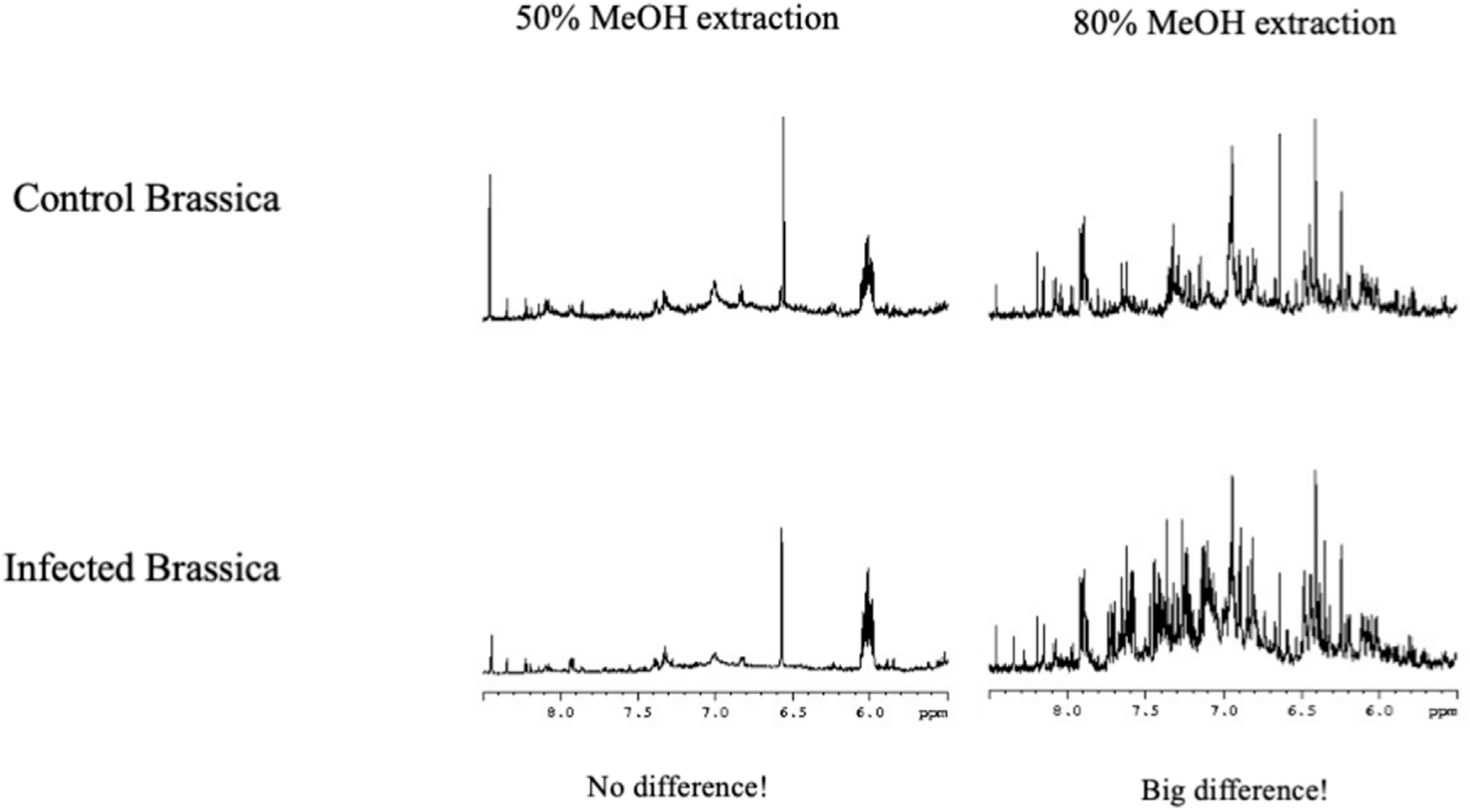
Difference between NMR metabolomics data depending on the extraction solvent. Control *Brassica nigra* and infected *Brassica nigra* leaves were extracted using two different solvents containing 50% MeOH **(A)** and 80% MeOH **(B)**. There was less differences between control and infected leaves when extracted with 50% MeOH. However when it is extracted using 80% MeOH, a big difference could be found in both extracts, showing the choice of extraction solvent is important (Verpoorte et al., 2007).

Metabolomics is used to identify markers for certain conditions, by comparing metabolomes of different materials, and identifying what signals correspond with what condition. Using a polar solvent to extract biomaterials means that certain compounds are poorly dissolved. Consequently, the signals of these compounds represent the peak height of a saturated solution of those compounds and will be similar for all samples. By only focusing on differences between metabolomes one might miss markers that are poorly soluble. From the fact that certain signals not seem to change, no conclusions can be drawn. Changing the extraction solvent may have a great impact on the visible metabolome and new markers might become visible (see Figure 7) (Verpoorte et al., 2007).

Another example of this problem is in the extraction of a given weight of sample with different amounts of solvent, e.g., 2 ml or 10 ml solvent. The total amount of a poorly dissolved compound differs a factor 5 between these extracts. When these extracts are taken to dryness and then redissolved in a well-defined amount of another solvent in which the compound is very well soluble, you will find a large difference for the amount detected in the two samples, though they could be similar quantities in the extracted materials. In liquid chromatography the choice of mobile phase is crucial for the separation. The pH is an important factor to keep in mind, as spectral data may be quite different for a compound when measured at different pH. An example is magnoflorine, a quaternary alkaloid. For many years there where two alkaloids mentioned in the literature, *N,N*-dimethyllindcarpine and magnoflorine, but finally it turned out that is was one and the same compound, with quite different spectral data if measured at high or low pH (Stermitz et al., 1980). Schripsema and colleagues (1986) used the pH effect as a tool for structure elucidation, as with trifluoroacetic acid one could deconvolute NMR spectra with a lot of overlapping signals, because the pH strongly affected the shift of protons close to nitrogen atoms. In identification of compounds by spectral data of LC-MS or GC-MS one should keep in mind that the pH of the injected sample may greatly affect retention and MS-fragmentation.

Finally, one other experience we want to share is about salicylic acid. An interesting compound as it is a signal compound that affects the metabolome of plants. We noted in literature that reported recoveries of salicylic acid varied from 30–60%. Un unacceptable difference, that will invalidate any conclusions when measuring this compound. We studied this in some detail and found that the problem is that salicylic acid is volatile. When taking an extract to full dryness, it disappears completely (Verberne et al., 2002). By adding a small amount of sodium hydroxide the evaporation was avoided, and high reproducible recovery was obtained.

## 6 Conclusion

Metabolomes of biological materials are complex, because of the large number of compounds with a wide range of polarities and concentrations. In preparing samples for metabolomic analysis extraction with organic solvents is a common step. These solvents may interact with various analytes through chemical reactions. Also contaminations in the solvents may be involved in the formation of artifacts. Particularly hydroxyl-, aldehyde-, keto-, carboxyl-, ester-, and amine functional groups are involved in the artifact formation. Oxidation, esterification, hydrolysis, glycolysis are common reactions that may occur in the preanalytical steps of the sample preparation. Considering the problems with some of the classic organic solvents, in terms of artifacts formation, their toxicity and their ecological damage, future research should be focused on developing novel green solvents for analytical chemistry, like the use of ionic liquids or natural deep eutectic solvents (Dai et al., 2013). In the LC analytical steps it is differences in injection solvent and mobile phase that are sources of artifacts, like distorted peaks or even double peak formation. Saturated solutions of poorly soluble compounds may hide markers. Finally the proper identification of compounds is a major hurdle, as it requires the full set of spectral data (UV, IR, MS, NMR), and methods for proving the full stereochemistry. Identification on the basis of UV, MS and retention is not sufficient. The development of a metabolomics analysis protocol should include a proper validation. For reproducible results the quality of all used chemicals and solvents should be controlled. For future reference, registration of the metadata from all steps of the protocol from collection to final chemometric analysis is essential.
